# Supplementary material for: Near-Hexaploid and Near-Tetraploid Aneuploid Progenies Derived from Backcrossing Tetraploid Parents Hibiscus syriacus × (H. syriacus × H. paramutabilis)
Source: Genes (Basel). 2022 Jun 6;13(6):1022. doi: 10.3390/genes13061022 (PMC9222940; doi:10.3390/genes13061022)
Supplement: Supplementary file 1 [file genes-13-01022-s001.zip › Supplement Table S1.pdf]

Supplement Table S1

Table S1. Progeny number and male-parent-confirmation primers for each cross combination between *Hibiscus*

Female parent	Male parent	Number of seedlings	Primer (forward) *	Primer (reverse)	Reference (primer)
H. syriacus 'Lavender Chiffon'	'Lohengrin'	24	5′-CACACACACACAGT -3′	-	[1]
H. syriacus 'Blushing Bride'	'Lohengrin'	65	5′-DHBCGACGACGACGA-3′	-	[2]
H. syriacus 'Pink Chiffon'	'Lohengrin'	15	5′-CACACACACACAGT-3′	-	[1]
H. syriacus 'Raspberry Smoothie'	'Lohengrin'	7	5′-TGAGTCCAAACCGGATA-3′	5′-GACTGCGTACGAATTCAA-3′	[3]
H. syriacus 'Strawberry Smoothie'	'Lohengrin'	66	5′-TGAGTCCAAACCGGATA-3′	5′-GACTGCGTACGAATTCAA-3′	[3]
H. syriacus 'White Chiffon'	'Lohengrin'	56	5′-CTCCTCCTCCTCCTCGC-3′	-	[1]
H. syriacus 'Blue Chiffon	'Resi'	19	5′-TGAGTCCAAACCGGTCG-3′	5′-GACTGCGTACGAATTaac-3′	[3]
H. syriacus 'Blushing Bride	'Resi'	18	5′-DHBCGACGACGACGA-3′	-	[2]
H. syriacus 'Lavender Chiffon	'Resi'	11	5′-CTCCTCCTCCTCCTCGC-3′	-	[1]
H. syriacus 'Raspberry Smoothie	'Resi'	1	5′-TGAGTCCAAACCGGATA-3′	5′-GACTGCGTACGAATTCAA-3′	[3]
H. syriacus 'White Chiffon	'Resi'	10	5′-CTCCTCCTCCTCCTCGC-3′	-	[1]
'Lohengrin'	*H. syriacus* 'Blushing Bride'	2	5′-DHBCGACGACGACGA-3′	-	[2]
**Total**		**294**			

*ISSR is a single primer marker

syriacus cultivars and the interspecific hybrids 'Lohengrin', 'Tosca', and 'Resi'.

1. Khafaga, A. Molecular genetic identification of some Egyptian *Hibiscus* samples. *The Journal of American Science* **2013**, *9*, 28-35.
2. Satya, P.; Karan, M.; Kar, C.; Mahapatra, A.; Mahapatra, B. Assessment of molecular diversity and evolutionary relationship of kenaf (*Hibiscus cannabinus* L.), roselle (*H. sabdariffa* L.) and their wild relatives. *Plant systematics and evolution* **2013**, *299*, 619-629.
3. Yu, C.; Yin, Y.; Creech, D.L.; Lu, Z.; Xu, J. Morphological characters and SRAP analysis of two hybrids between *Hibiscus dasycalyx* and *Hibiscus* 'Moy Grande'. *Sci Hortic-Amsterdam* **2016**, *198*, 118-124.
