# Supplementary material for: Near-Hexaploid and Near-Tetraploid Aneuploid Progenies Derived from Backcrossing Tetraploid Parents Hibiscus syriacus × (H. syriacus × H. paramutabilis)
Source: Genes (Basel). 2022 Jun 6;13(6):1022. doi: 10.3390/genes13061022 (PMC9222940; doi:10.3390/genes13061022)
Supplement: Supplementary file 1 [file genes-13-01022-s001.zip › Supplemental Table S2.pdf]

Supplemental Table S2. Genome sizes and groupings of interspecific hybrid *Hibiscus* cultivars, Lohengrin, Tosca, and Resi and BC_1F_1 hybrids [A]

Plant ID – cultivar name or Accession number	Mean genome size (pg) ± SE	repeat	Predicted plod level	Groups[B]
H2015-016-11	7.21 ± 0.09	3	6*x*	a
H2015-016-03	7.16 ± 0.08	3	6*x*	ab
H2015-109-01	7.09 ± 0.08	3	6*x*	abc
H2015-016-02	7.07 ±0.03	3	6*x*	abc
H2015-016-01	7.05 ±0.03	3	6*x*	abc
H2015-019-08	7.05 ± 0.01	3	6*x*	abc
H2015-016-05	7.04 ± 0.08	3	6*x*	abc
H2015-016-08	7.03 ± 0.01	3	6*x*	abc
H2015-024-05	7.02 ± 0.05	3	6*x*	bc
H2015-024-08	7.00 ± 0.03	3	6*x*	bc
H2015-024-01	6.98 ± 0.02	3	6*x*	c
H2015-024-07	6.97 ± 0.03	3	6*x*	c
H2015-019-09	6.96 ± 0.05	3	6*x*	c
H2015-017-05	6.94 ± 0.07	3	6*x*	c
H2015-047-05	4.95 ± 0.13	3	4*x*	d
H2015-122-03	4.87 ± 0.05	3	4*x*	de
H2015-081-01	4.83 ± 0.02	3	4*x*	def
H2015-052-02	4.83 ± 0.05	3	4*x*	def
H2015-062-08	4.83 ± 0.04	3	4*x*	def
H2015-043-07	4.83 ± 0.08	3	4*x*	def
H2015-122-01	4.82 ± 0.03	3	4*x*	def
H2015-122-02	4.81 ± 0.04	3	4*x*	def
H2015-052-12	4.80 ± 0.06	3	4*x*	defg
H2015-052-X2	4.79 ± 0.03	3	4*x*	defg
H2015-060-23	4.78 ± 0.05	3	4*x*	defg
H2015-064-10	4.76 ± 0.03	3	4*x*	efgh
H2015-108-02	4.76 ± 0.07	3	4*x*	efgh
H2015-046-09	4.76 ± 0.05	3	4*x*	efgh
H2015-072-16	4.75 ± 0.04	3	4*x*	efgh
H2015-061-03	4.75 ± 0.01	3	4*x*	efgh
H2015-043-06	4.75 ± 0.02	3	4*x*	efgh
H2015-076-08	4.75 ± 0.04	3	4*x*	efgh
H2015-044-07	4.73 ± 0.07	3	4*x*	efgh
H2015-052-05	4.72 ± 0.02	3	4*x*	efgh
H2015-029-02	4.72 ± 0.03	3	4*x*	efgh
H2015-031-04	4.72 ± 0.00	3	4*x*	efgh
H2015-102-03	4.66 ± 0.06	3	4*x*	fgh
H2015-068-01	4.63 ± 0.09	3	4*x*	gh
'Resi'	4.63 ± 0.08	3	4*x*	gh
'Fiji'	4.63 ± 0.05	3	4*x*	gh
'Lohengrin'	4.58 ± 0.03	3	4*x*	h

[A] A total of 18 progenies of 'Resi' and of 19 progenies of 'Lohengrin' were randomly chosen from a population that has been selected by general plant growth vigor.
[B] Grouped by Tukey's Honest Significant Difference (HSD) test ($\alpha = 0.05$) for mean genome size comparisons
